# The Rise of Mechanobiology for Advanced Cell Engineering and Manufacturing

**DOI:** 10.1002/adma.202501640

**Published:** 2025-06-27

**Authors:** Huan Ting Ong, M Sriram, Hepi Hari Susapto, Yixuan Li, Yuan Jiang, Nicolas H. Voelcker, Jennifer L. Young, Andrew W. Holle, Roey Elnathan

**Affiliations:** ^1^ Mechanobiology Institute National University of Singapore Singapore 117411 Singapore; ^2^ Monash Institute of Pharmaceutical Sciences Monash University 381 Royal Parade Parkville VIC 3052 Australia; ^3^ Melbourne Centre for Nanofabrication Victorian Node of the Australian National Fabrication Facility 151 Wellington Road Clayton VIC 3168 Australia; ^4^ Department of Biomedical Engineering College of Design and Engineering National University of Singapore Singapore 117583 Singapore; ^5^ School of Medicine Faculty of Health Deakin University Waurn Ponds VIC 3216 Australia; ^6^ Institute for Frontier Materials Deakin University Geelong Waurn Ponds campus Waurn Ponds VIC 3216 Australia; ^7^ The Institute for Mental and Physical Health and Clinical Translation School of Medicine Geelong Waurn Ponds Campus Deakin University Melbourne VIC 3216 Australia

**Keywords:** biomaterials, cell engineering, cell manufacturing, confinement, extracellular matrix, intracellular delivery, mechanobiology

## Abstract

The rise of cell‐based therapies, regenerative medicine, and synthetic biology, has created an urgent need for efficient cell engineering, which involves the manipulation of cells for specific purposes. This demand is driven by breakthroughs in cell manufacturing, from fundamental research to clinical therapies. These innovations have come with a deeper understanding of developmental biology, continued optimization of mechanobiological processes and platforms, and the deployment of advanced biotechnological approaches. Induced pluripotent stem cells and immunotherapies like chimeric antigen receptor T cells enable personalized, scalable treatments for regenerative medicine and diseases beyond oncology. But continued development of cell manufacturing and its concomitant clinical advances is hindered by limitations in the production, efficiency, safety, regulation, cost‐effectiveness, and scalability of current manufacturing routes. Here, recent developments are examined in cell engineering, with particular emphasis on mechanical aspects, including biomaterial design, the use of mechanical confinement, and the application of micro‐ and nanotechnologies in the efficient production of enhanced cells. Emerging approaches are described along each of these avenues based on state‐of‐the‐art fundamental mechanobiology. It is called on the field to consider mechanical cues, often overlooked in cell manufacturing, as key tools to augment or, at times, even to replace the use of traditional soluble factors.

## Graphical abstract

With the growing demand for cell‐based therapies, efficient cellular engineering is crucial. This review calls for greater recognition of mechanobiology principles applied through advanced biomaterial designs, mechanical confinement, and highlights recent advances using micro/nanotechnologies to enhance cell manufacturing. Challenges and opportunities are also discussed to encourage further innovation in advanced cell engineering.

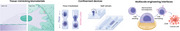

## 1 Cell Engineering and Manufacturing Goals

Substantial progress in cell engineering and manufacturing has paved the way for transformative medical therapies capable of precisely targeting, repairing, and regenerating tissues.^[^ ^1^ ^]^ While the clinical success of these engineered cell products has set a new benchmark in therapeutic care, it has also driven the field into new interdisciplinary terrains. Researchers are now focused on developing more refined cellular products that offer enhanced efficacy while addressing the issues of simplifying manufacturing complexities and reducing costs.

Cell engineering and manufacturing continue to pose major challenges for translational biomedical research.^[^ ^2^ ^]^ The difficulty lies in manipulating cells to transform them without causing irreversible loss of function. This complexity is further compounded by the need to scale up cell production, standardize manufacturing protocols, and adhere to strict regulatory frameworks, which are critical steps for engineering and producing next‐generation cell products with therapeutic potential. Achieving these goals requires more than a deep understanding of cell biology and genetic or biochemical approaches; it also demands the development of advanced tools and techniques, along with the ability to regulate cellular processes with high specificity and reproducibility.^[^ ^3^ ^]^ This includes leveraging emerging biomaterial and nanoscale routes and confinement mechanobiology to optimize cell behavior and functionality during engineering and manufacturing.

As the field advances, significant challenges in cell engineering and manufacturing have also emerged, particularly in translating these innovations into scalable, reproducible, and regulatory‐compliant therapeutic cellular products. Overcoming these hurdles is essential in the field of cell engineering, which has revolutionized contemporary medicine by using the unique properties of cells as a “living drug”. This concept, once considered visionary, has become a tangible reality, enabling the development of therapies that can precisely target, repair, and regenerate tissues.

The overarching goal of cell engineering is to address a wide range of diseases by engineering and manufacturing cells with specific therapeutic functions. Researchers have focused on a diverse array of cell types, primarily *stem cells* and *immune cells*, in targeted therapeutic applications. Stem cells are primarily used for their regenerative potential to replace and repair damaged tissues, while immune cells are designed to detect and eliminate diseased cells, such as in cancer therapy.

Among all cell types, stem cells and immune cells are the key platforms for innovation in ex vivo cell engineering and manufacturing. Multipotent somatic stem cells such as hematopoietic stem cells (HSCs) and mesenchymal stem/stromal cells (MSCs) are particularly significant: they differentiate into tissue‐specific and lineage‐restricted cell types and represent the first generation of stem cells to achieve clinical application. For instance, bone marrow transplants containing HSCs have been used for more than 60 years to treat patients with blood cancers, making HSCs the most widely used stem cell type in clinical practice.^[^ ^4^ ^]^

Similarly, immune cells such as CAR T cells have demonstrated transformative potential in oncology.^[^ ^5^ ^]^ By leveraging their natural ability to migrate to and target tumors, CAR T cells have become a powerful therapeutic tool for combating certain cancer types. Building on this success, researchers are exploring new avenues to expand the application of engineered cell therapies. These include engineering T cells with immunosuppressive properties to treat inflammatory and autoimmune conditions, as well as designing T cells with tissue‐sensing capabilities for localized delivery of therapeutic payloads. Such innovations highlight the versatility of engineered immune cells and their potential to address a broader spectrum of diseases beyond cancer.^[^ ^6^ ^]^

In addition to their therapeutic roles, engineered cells are being used as living factories to produce biologics, including antibodies and therapeutic proteins, which are crucial for treating various diseases. For example, MSCs are being engineered to secrete therapeutic proteins such as vascular endothelial growth factors (VEGFs) to promote angiogenesis and tissue repair.^[^ ^7^ ^]^ And engineered T cells are being employed to produce cytokines, such as interleukin‐2 (IL‐2), to enhance immune responses against cancer.^[^ ^8^ ^]^

Mechanobiology has emerged as a critical lens for advancing cell engineering and manufacturing.^[^ ^9^ ^]^ Cells interact with the matrix through the binding of transmembrane adhesion receptors (e.g., integrins) to specific extracellular matrix (ECM) ligands, causing integrin clustering and the assembly of focal adhesions (FAs) inside the cell. FAs play a central role in sensing, transmitting, and converting mechanical cues into biochemical signals that initiate downstream signaling processes (mechanotransduction) and ultimately lead to gene transcription.^[^ ^10^ ^]^ Recent findings have also identified certain microRNAs (miRNAs), small non‐coding RNAs, as mechanosensitive regulators of gene expression, acting through a miRNA‐cytoskeletal‐matrix‐actin (CAM) mRNA regulatory network post‐transcriptionally.^[^ ^11^ ^]^ Mechanobiological principles thus offer novel insights into how the physical microenvironment (e.g., ECM properties and dynamic forces) regulates fundamental cellular processes like migration, proliferation, and differentiation. Emerging evidence suggests that these cues can be systematically tuned to optimize cell performance, improve therapeutic efficacy, and standardize manufacturing protocols. As the demand for scalable, reproducible, and efficient cell‐based therapies grows, integrating biomaterial engineering, nanotechnology, and mechanobiological principles into cell engineering promises to unlock new frontiers in precision medicine. This review explores how mechanobiology is shaping the future of cell engineering, with a particular focus on its role in controlling cell behavior, enabling large‐scale production of functional cells, and enhancing therapeutic outcomes.

## 2 Tissue‐Mimicking Biomaterials for Cell Engineering

The majority of current iPSC reprogramming and stem cell differentiation protocols use conventional cell culture strategies on 2D stiff tissue culture plastic substrates coated with a thin ECM layer, such as Geltrex, Matrigel, vitronectin, gelatin, or laminin.^[^ ^12^, ^13^ ^]^ These simple culture conditions have been implemented due to limitations arising from costs, scale‐up, and availability, yet their efficiency and efficacy are lacking. Engineering innovations have emerged in recent years to address key challenges in cellular therapies that result from conventional cell culture.

By leveraging biomaterials to reconstruct properties of the native cell niche in vitro and applying principles of mechanobiology, cell fate and function can be controlled.^[^ ^14^ ^]^ In one of the earliest examples, culturing human mesenchymal stem cells on soft, elastic hydrogels mimicking tissue‐specific stiffnesses demonstrated the ability of biophysical properties alone to direct differentiation to the respective tissue type of each stiffness.^[^ ^15^ ^]^ Numerous studies have since focused on designing biomaterials to mimic a multitude of ECM features in order to direct cellular behavior in vitro.^[^ ^14^, ^16^ ^]^

**Figure 1:**
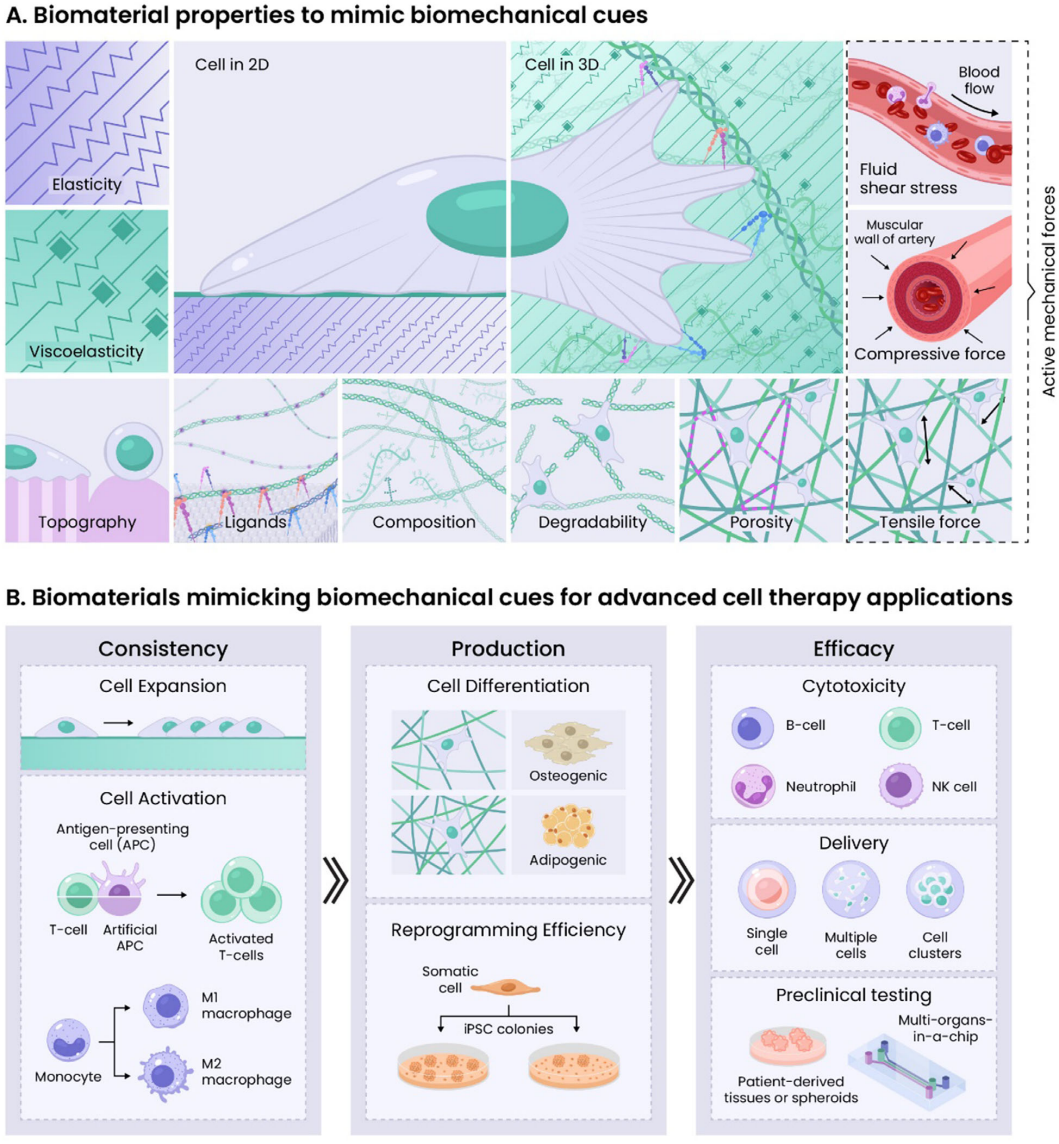
Biomaterial strategies for cell manufacturing: A) A wide variety of extracellular cues can be incorporated into biomaterials design to direct cellular function, including biophysical cues (elasticity, viscoelasticity, degradability), architectural cues (topography, ligand organization, porosity), biochemical cues (composition), and active cues (tensile force, compressive force, shear stress). B) These cues can be used for improving cell growth consistency (better cell expansion or activation), production (enhanced stem cell differentiation or reprogramming efficiency), and efficacy (improved cytotoxicity, delivery, or testing outcomes).

Biomaterials, in 2D or 3D, can be engineered to provide physiologically relevant *biochemical cues* (e.g., composition), *architectural cues* (e.g., ligand organization, topography, porosity), and *biophysical cues* (e.g., elasticity, viscoelasticity, degradability).^[^ ^17^ ^]^ *Active dynamic cues* (e.g., compressive forces, tensile forces, shear stress) that mimic time‐dependent biological processes can also be incorporated into material systems^[^ ^18^ ^]^ (**Figure** **1**A). These physiologically relevant cues can influence cell‐matrix interactions, which in turn influence cellular behavior and phenotype via mechanotransduction. Ultimately, these cues can be leveraged for improving cell growth, consistency, production, and efficiency in cell therapies (Figure 1B).

### 2.1 Tuning Elasticity to Mimic Tissue Stiffness

Substrate stiffness has been shown to influence cell fate and function in numerous in vitro studies via the process of mechanotransduction.^[^ ^19^ ^]^ Therefore, tuning the Young's modulus of biomaterials to match physiologically relevant tissue stiffness values can help prime cells toward a desired phenotype in cell engineering and manufacturing.

For stem cells, classical approaches employ chemical cues to either maintain stemness or promote differentiation, and are often resource‐ and time‐intensive. However, it has been shown that substrate stiffness alone can direct stem cells since actomyosin contractility is known to promote stem cell differentiation. For example, in the well characterized 2D polyacrylamide (PA) hydrogel system, softer matrices, mimicking brain elasticity (Young's modulus, *E* ≈1 kPa) promoted mesenchymal stem cell (MSC) neurogenesis, while stiffer matrices, mimicking pre‐calcified bone (*E* ≈35 kPa), drove osteogenesis.^[^ ^20^ ^]^ Polyethylene glycol (PEG)‐based hydrogel systems are often utilized due to their tunability and biocompatibility, and when MSCs were cultured on a stiffer substrate (*E* ≈40 vs ≈5 kPa), they stimulated osteogenesis. Similarly in hematopoietic stem cells (HSCs), culture on soft Gelatin Methacryloyl (GelMA) hydrogels of *E* ≈4 kPa led to erythroid lineages, while those cultured on stiff *E* ≈44 kPa substrates favored other lineages. This is an important consideration in therapeutic approaches due to the fact that the mechanical properties of HSC bone marrow niches increase with age in mice, from *E* ≈110 Pa in 2‐month‐old mice to *E* ≈310 Pa in 18‐month‐old mice.^[^ ^21^ ^]^

Somatic cell reprogramming to induced pluripotent stem cells (iPSCs) has emerged as a standardized approach to create cell sources for expansion, reducing heterogeneity in downstream manufacturing and improving patient‐specificity. Both the reprogramming step and subsequent differentiation process are sensitive to substrate stiffness, which is also specific to the starting cell type. MSC reprogramming has been shown to be most efficient on polydimethylsiloxane (PDMS) hydrogels of *E* ≈1.5 kPa^[^ ^17b^ ^]^ while for fibroblasts, systematic screening of PA hydrogel stiffnesses ranging from *E* ≈1 kPa to 1.3 MPa found the highest reprogramming efficiency on *E* ≈100 kPa.^[^ ^22^ ^]^ After the generation of iPSCs, their differentiation also toward specific lineages are also dependent on mechanics, with substrate stiffness being harnessed to generate MSCs,^[^ ^23^ ^]^ neuronal progenitors,^[^ ^12^, ^24^ ^]^ cardiomyocytes,^[^ ^25^ ^]^ platelets,^[^ ^26^ ^]^ CAR T cells,^[^ ^13^, ^27^ ^]^ or macrophages.^[^ ^13b^ ^]^

Aside from stem cells, adoptive cell therapies have increasingly employed a combinatorial approach, leveraging both biomaterial and genetic engineering to improve therapeutic efficacy and safety. On the biomaterial side, increasing substrate stiffness (from *E* ≈11 kPa to *E* ≈323 kPa) has been shown to polarize macrophages toward a proinflammatory phenotype (M1), which in turn influences their phagocytic activity and migration.^[^ ^19^, ^28^ ^]^

T‐cell functions, including proliferation, activation, and cytotoxicity, are also substrate stiffness dependent.^[^ ^29^ ^]^ For example, T cells activated on PA gels of intermediate stiffness (*E* ≈25 kPa) secreted more IL‐2 compared to those on softer or stiffer gels (*E* ≈5 kPa or *E* ≈110 kPa, respectively).^[^ ^30^ ^]^ Artificial antigen‐presenting cells (aAPCs), often in the form of antibody‐coated polystyrene‐based beads such as Dynabeads or MACSiBeads, are commonly used in T cell activation strategies.^[^ ^31^ ^]^ The effectiveness of aAPC‐mediated T‐cell activation significantly impacts transfection efficiency,^[^ ^32^ ^]^ expansion,^[^ ^33^ ^]^ and differentiation in CAR T‐cell production. B cells have been cultured on 2D PDMS substrates of variable stiffness (ranging from *E* ≈20 kPa to ≈1100 kPa) to regulate their differentiation potential for specific functionalities, including receptor accumulation, proliferation, class switching, and antibody production.^[^ ^34^ ^]^

While all these examples were carried out in 2D, 3D approaches can more faithfully recapitulate the in vivo microenvironment. In vitro studies have identified dimensionality‐specific stem cell mechanotransduction, with yes‐associated protein (YAP) nuclear translocation most pronounced on stiff 2D substrates (e.g., PA gels). In 3D, cell volume plays a greater role, with stiff 3D matrices (e.g., gelatin methacrylate) restricting cell spreading, thereby downregulating YAP translocation.^[^ ^9^, ^35^ ^]^ Further, Stiff 3D matrices have been shown to drive mechanotransduction through mechanisms independent of YAP.^[^ ^19^, ^36^ ^]^ Beyond stem cell differentiation, 3D elastic materials can be used for encapsulating cells for targeted delivery. For instance, soft PEG‐PLA based biomaterial with an elastic modulus (*E* <1 kPa) can be used for delivering newly engineered CAR T cells to tumor sites, minimizing CAR T cell–exhaustion.^[^ ^37^ ^]^ Similarly, soft hydrogels such as Arg‐Gly‐Asp (RGD)‐alginate (*E* ≈2 kPa), can be employed for the delivery of MSC by providing a defined microenvironment post‐transplantation.^[^ ^38^ ^]^

### 2.2 Viscoelasticity Better Recapitulates In Vivo Tissue Mechanics

While elastic materials have elucidated important mechanobiological phenomena, tissues are known to be viscoelastic, with an estimated loss modulus (*G“”)* of ≈10–20% of their elastic modulus.^[^ ^39^ ^]^ This fluid‐like property allows tissue to dissipate cell‐induced forces, which in turn regulates cell behavior and ECM remodeling.^[^ ^40^ ^]^ The viscoelastic properties of tissues vary across the body, with a loss modulus of *G“”*≈10^3^ Pa in the brain to *G“”* ≈10^9^ Pa in the bone.^[^ ^40^ ^]^ These viscoelastic properties also influence stem cell fate through cellular volume expansion, as shown by studies using engineered viscoelastic alginate hydrogels with tunable stress relaxation.^[^ ^35^ ^]^ Specifically, cell spreading, migration, and proliferation are enhanced in hydrogels with a higher loss modulus (i.e., faster stress relaxation).^[^ ^41^ ^]^ In addition, human iPSCs maintained pluripotency when encapsulated in 3D viscoelastic alginate‐based hydrogels longer than the traditional Matrigel,^[^ ^42^ ^]^ and more viscoelastic hydrogels provide cell fate and patterning cues via increased YAP nuclear translocation in organoid cultures.^[^ ^43^ ^]^

In a 2D viscoelastic alginate hydrogel system, it was demonstrated that MSC migration improved on soft substrates (*E* ≈2 kPa) with faster stress relaxation (≈100 s for the normalized stress to decrease to half its peak value).^[^ ^41^ ^]^ In contrast, minimal migration was observed on gels with slower stress relaxation (≈2200 s), even when the stiffness was identical (*E* ≈2 kPa). This suggests that efficient cell migration on soft substrates is highly dependent on viscoelasticity.^[^ ^41^ ^]^ While this is largely adopted in tissue regeneration applications, viscoelastic hydrogels have also been proven valuable for activating non‐adherent cells such as CAR T cells^[^ ^44^ ^]^ by mimicking the natural T cell‐APC interactions.^[^ ^31^ ^]^ For example, antibody‐functionalized alginate‐based microgels with varying viscoelastic properties were used as aAPCs and demonstrated enhanced T‐cell activation compared to conventional stiff Dynabeads.^[^ ^31^, ^44^ ^]^

Using various non‐covalent or reversible linkages, viscoelastic hydrogels can be engineered to facilitate cell force‐mediated matrix remodeling similar to native ECM.^[^ ^45^ ^]^ The matrix deformation enables the formation of ligand clustering, which locally mediates cell adhesion, contractility, and spreading on soft viscoelastic substrates, similar to cells cultured on stiff elastic substrates.^[^ ^46^ ^]^ In 3D cultures, cells can more readily remodel the matrix with less resistance compared to static elastic hydrogels, thereby promoting cell mechanotransduction.^[^ ^9^, ^47^ ^]^ Moreover, fibrous ECM and cytoskeletal proteins also exhibit non‐linear elasticity, such as strain stiffening, which is crucial for resisting deformation.^[^ ^48^ ^]^ While viscoelastic hydrogels with stress relaxation have been extensively studied for their influence on cellular behavior, strain stiffening remains understudied. It has been reported that MSCs cultured in a polyisocyanopeptide hydrogel with strain‐stiffening properties promote osteogenic differentiation through the microtubule‐associated protein DCAMKL1.^[^ ^49^ ^]^ Additionally, stress stiffening facilitates the formation of protrusions in mesenchymal stem cells (MSCs) with mature focal adhesion and high cell contractility.^[^ ^45a^ ^]^ By integrating both stress relaxation and strain stiffening, engineered hydrogels may more accurately replicate native ECMs, providing a promising platform for enhancing cell manufacturing.

### 2.3 Incorporation of Active Mechanical Cues to Direct Cells

Cells in vivo experience time‐dependent forces, including tension, compression, and shear stress, which have been shown to influence both cell fate and function.^[^ ^18^, ^50^ ^]^ For example, immune cells experience fluid shear stress as they circulate through blood vessels. This dynamic mechanical environment plays a critical role in cellular behavior and has inspired efforts to incorporate such mechanical cues into in vitro cell manufacturing systems. Applying shear stress to iPSC‐derived platelets has been shown to enhance their differentiation compared to static cultures.^[^ ^26^ ^]^ Similarly, T‐cell activation, monocyte proinflammatory cytokine secretion, and cytotoxicity of Natural Killer (NK) cells were all significantly improved when exposed to fluid shear stress, mediated via mechanosensitive ion channel PIEZO1.^[^ ^9^, ^18^, ^51^ ^]^ These examples underscore the importance of incorporating mechanical cues like shear stress to modulate cellular responses in therapeutic applications.

Compressive stress has also been explored as a key regulator of stem cell behavior. Application of uniaxial cyclic compressive stress to MSC spheroids embedded in viscoelastic, RGD‐functionalized 3D alginate hydrogels resulted in mechanical loading induced pro‐inflammatory and pro‐angiogenic secretions.^[^ ^18a^ ^]^ Tensile forces, another physiologically relevant active mechanical cue, have been shown to promote osteogenic differentiation in MSCs.^[^ ^18^, ^52^ ^]^ Strategies to generate tensile force in vitro include the use of magnetic nanoparticles to apply forces mimicking tensile strains,^[^ ^52^ ^]^ uniaxial cyclic stretch to MSC monolayers cultured on elastic PDMS cell chambers,^[^ ^18c^ ^]^ and equibiaxial stretch using BioFlex cell culture plates.^[^ ^18d^ ^]^

### 2.4 Biochemical and Architectural ECM Cues to Drive Cells

Biochemical and architectural cues from the ECM, including composition, ligand organization, ligand density, topography, degradability, and porosity, can be used to elicit specific cell‐matrix signalling pathways for cell manufacturing strategies. The composition of the ECM (e.g., proteins, enzymes, growth factors, polysaccharides, and glycoproteins)^[^ ^53^ ^]^ orchestrates a range of biological processes, including cell attachment, migration, blood vessel formation, T‐cell activation, and organogenesis.^[^ ^54^ ^]^ Biomaterial design of specific ECM components uses either synthetically produced bioactive moieties (e.g., peptides)^[^ ^55^ ^]^ or naturally‐derived bioactive moieties (e.g., decellularized ECM).^[^ ^56^ ^]^ Of the peptide‐derived moieties, RGD is the most ubiquitous^[^ ^57^ ^]^ for cell attachment and has been used to direct stem cell fate.^[^ ^58^ ^]^

Architectural cues can be designed at different length scales to direct stem cell fate. At the microscale, micropatterns are used to dictate cell morphology, such as surface roughness, electrospun nanofibers, shapes like circles, rectangles, and triangles, or nanoneedles of different geometries, have been shown to drive stem cell functions and behaviors such as differentiation, cell adhesion, and secretome production.^[^ ^59^ ^]^ At the nanoscale, mesoporous silica nanoparticle‐based biomaterials with tunable ligand density and spatial organization direct stem cell differentiation by influencing cell adhesion and spreading.^[^ ^60^ ^]^

The porosity of biomaterials can be tuned to improve nutrient and oxygen supply.^[^ ^14b^ ^]^ Microporous scaffolds have proven effective in reconstructing 3D tissue structures by facilitating cell‐matrix network formation and vascularization.^[^ ^61^ ^]^ In cell therapies, porous biomaterials are particularly useful for cell retention during delivery to the target site^[^ ^62^ ^]^ to direct differentiation,^[^ ^60^, ^63^ ^]^ and for the slow release of paracrine secretory factors in vitro.^[^ ^64^ ^]^ Biomaterials with tunable degradability have also been used for stem cell and organoid transplantation, where gradual degradation supports long‐term tissue regeneration.^[^ ^65^ ^]^ Degradability, which can also influence the material's viscoelastic properties over time, can be controlled by the incorporation of cell‐degrading peptides (e.g., MMP‐sensitive domains)^[^ ^66^ ^]^ or hydrolytic ester linkages.^[^ ^67^ ^]^

For immune cells, topographical and ligand features of biomaterials have also been utilized to influence their fate and function.^[^ ^68^ ^]^ The size of aAPCs affects T‐cell activation, with smaller microgels causing an increase in T‐cell expansion^[^ ^44^ ^]^ while aAPC ligand density has been shown to enhance activation^[^ ^69^ ^]^ and cytokine secretion of T cells.^[^ ^30^ ^]^ Specifically, increasing ligand density – from 0 to 6.4 ug cm^−2^ – boosted both T‐cell expansion and cytotoxicity.^[^ ^44^ ^]^ In other examples, microrod‐shaped aAPCs were shown to more efficiently activate and expand T cells compared to spherical Dynabeads.^[^ ^70^ ^]^ Additionally, a study using degradable APC‐mimicking scaffold in the shape of microrods demonstrated the ability to control T cell stimulation via the slow release of mitogenic factors.^[^ ^33^ ^]^

### 2.5 The Future of Advanced Biomaterial Strategies for Scalable Cell Manufacturing

#### 2.5.1 Innovations to Improve Tissue Mimicry

The next frontier in advanced biomaterial innovations for cell manufacturing lies in integrating combinatorial and dynamic ECM cues that can more precisely prime, instruct, and guide cellular behavior. Emerging strategies to influence cell behavior through mechanobiology include dynamic biomaterials that change stiffness over time,^[^ ^45^, ^71^ ^]^ materials capable of precisely modulating nano‐ligand presentation (e.g., using magnetic platforms),^[^ ^72^ ^]^ and those that apply specific 3D active mechanical forces to control cellular behavior.^[^ ^18^, ^52^, ^71^ ^]^ These advanced materials enable the construction of increasingly sophisticated, biomimetic microenvironments – potentially extending to multi‐organ systems integrated through microfluidic platforms,^[^ ^73^ ^]^ thus enabling systematic testing and direction of cell behaviors for tailored therapeutic applications. Breakthroughs in micro, nano, and biofabrication techniques such as dynamic biomaterials (also known as stimuli‐responsive hydrogels),^[^ ^74^ ^]^ melt electrowriting,^[^ ^75^ ^]^ 3D bioprinting,^[^ ^76^ ^]^ and micropillars^[^ ^77^ ^]^ (discussed in greater detail in section 4) offer unprecedented control over complex microenvironments that can precisely direct cellular behavior, fate, and function.

#### 2.5.2 Limitations in Clinical Translation Using Biomaterials

For scalable manufacturing with biomaterials, several additional factors should be addressed, in addition to promoting specific cell functions. For example, for efficient cell expansion and harvesting methods, 3D approaches might be useful in tissue engineering strategies, but the low throughput and high costs could limit their applicability in cell manufacturing. In these cases, degradable microbeads in bioreactors could be adopted.^[^ ^78^ ^]^ Equally important are delivery approaches, such as encapsulating cells in microgels to mitigate damaging shear stress during intravenous delivery,^[^ ^79^ ^]^ improving their survival and therapeutic efficacy.^[^ ^62^ ^]^ As clinical applications continue to advance, biomaterial strategies must also evolve to become more refined, personalized, and fit‐for‐purpose, thereby driving forward the next generation of precision‐engineered cell therapies.

## 3 High‐Throughput Mechanical Confinement Strategies in Cell Engineering

While tissue‐mimetic biomaterials presenting complex biological cues are constantly redefining the boundaries of cell engineering, the past decade has seen the emergence of simple, high‐throughput cell engineering strategies driven by a unique yet ubiquitous biophysical cue: mechanical confinement. Cells in the body continuously experience confinement via neighboring cells, the ECM, and surrounding tissue. Cells are also exposed to diverse levels of confinement when they migrate, crossing endothelial layers, interstitial spaces, and dense tissue ECM.^[^ ^81^ ^]^ In the past fifteen years, confinement‐induced cellular changes have been exploited in vitro to induce desired cell states, making confinement an attractive tool for large‐scale cell engineering. This is a biomimetic approach: confining cells to specific microscale geometries mimics their native ECM microenvironment, and allowing cells to migrate through confining microchannels mimics the in vivo confined migration that has been shown to affect a wide variety of cellular processes.

Numerous strategies have emerged to confine cells in 1D, 2D, or 3D spaces (depending slightly on how dimensional spaces are defined), establishing an enormous source of knowledge on how different cell types, including stem cells, immune cells, and cancer cells, respond to different levels of confinement.^[^ ^82^ ^]^ The knowledge gained from these studies offers novel solutions to problems associated with cancer treatment, regenerative medicine, and other biomedical applications. However, for cell engineering in clinical applications, high‐throughput strategies with short processing times and high accuracy are needed to efficiently apply mechanical confinement in a controlled manner to cells of interest. Most current approaches use PDMS‐based microfluidic confinement devices with constricting microchannels, due to their low cost, flexibility, and ease of use. These devices enable the fabrication of highly controlled and reproducible confinement geometries, allowing precise manipulation of mechanical and spatial cues at the single‐cell level. They are particularly well‐suited for high‐throughput applications, enabling parallel processing and screening of large numbers of confined microenvironments.^[^ ^83^ ^]^ Given these advantages, this section will focus specifically on microfluidic confinement devices as tools for cell engineering and highlight how microfluidic confinement devices offer both fundamental insights and translational potential in biomedical research.

**Figure 2:**
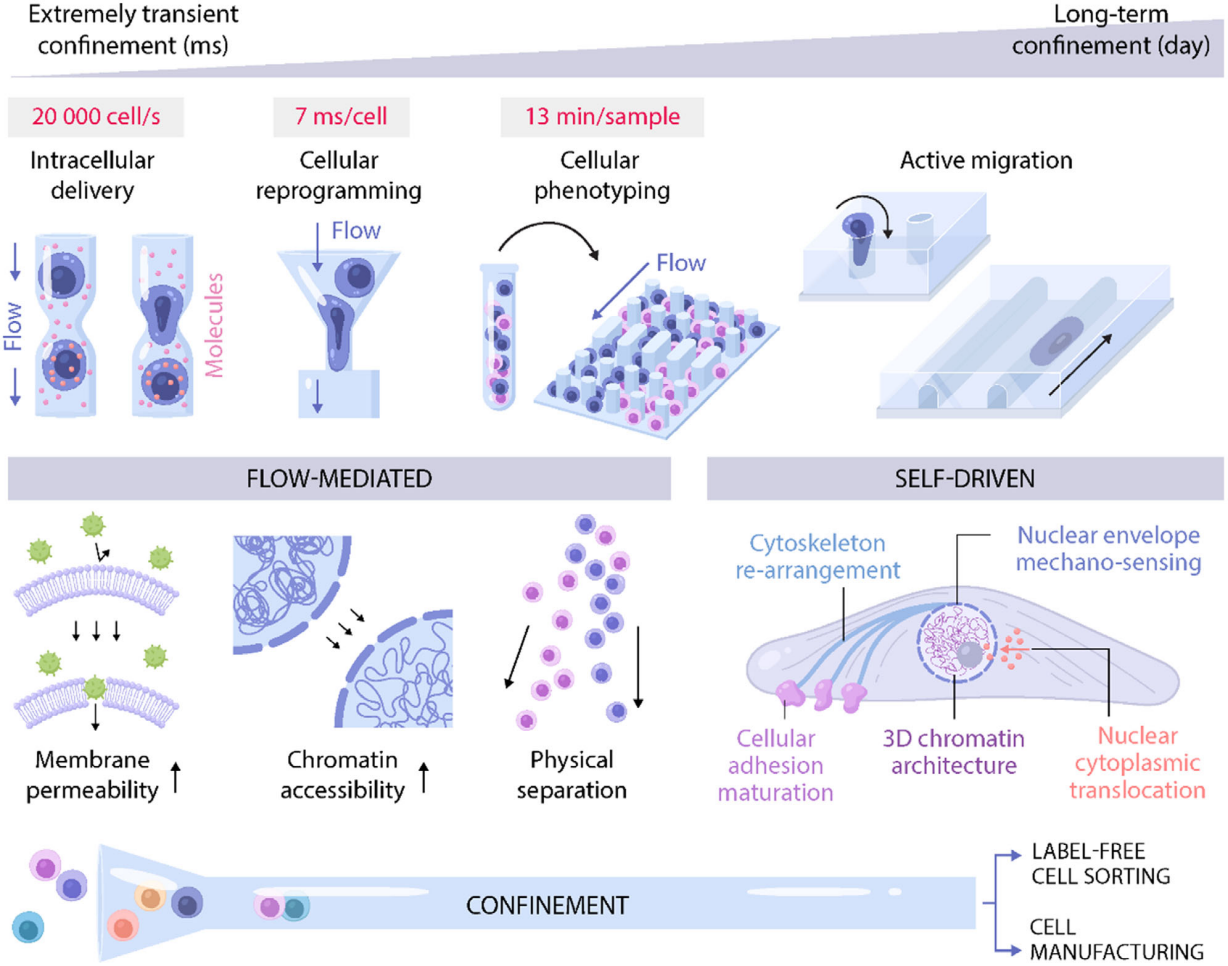
Squeezing into shape: the use of both passive, flow‐mediated (left) and active, self‐driven (right) confinement ultimately results in cell deformation, allowing for precise, high throughput control of confinement mechanobiology.

Microfluidic confinement devices fall under one of two categories based on how it present confining spaces to cells (**Figure** **2**). The first category is *flow‐mediated confinement devices*, which contain microchannels that are analogous to log flumes, propelling cells through a physically constricting environment via fluid flow, resulting in transient exposure to specific levels of confinement.^[^ ^84^ ^]^ The second category is *self‐driven confinement devices* which contain microchannels through which cells actively migrate, without any external fluid flow, either in the presence or absence of a chemoattractant.^[^ ^85^ ^]^ Both flow‐mediated and self‐driven confinement devices have distinct effects on cell behavior and state without the need for soluble factors, making them attractive tools for cell engineering and other biomedical applications.

### 3.1 Flow‐Mediated Confinement Devices

Flow‐mediated confinement devices are by definition, high throughput, owing to the high speeds at which cells “ride” the fluid current through the confining microchannels. When cells pass through these confining spaces, they undergo rapid cell and nuclear deformation^[^ ^84b^ ^]^ develop transient pores on their plasma membrane,^[^ ^86^ ^]^ and experience changes in their volume.^[^ ^87^ ^]^ These cellular dynamics have only recently been exploited to develop confinement‐mediated high‐throughput cell‐engineering techniques (Figure 2).

#### 3.1.1 Flow‐Mediated Confinement Devices for Intracellular Cargo Delivery

Intracellular delivery of exogenous cargo has always been a grand challenge in cell engineering. This cargo, whether it be biological, chemical, or physical in nature, is designed to elicit specific effects, behaviors, or states in target cells, and thus control over delivery is control of the cells themselves. Matching this diversity of cargo, a wide array of biological, chemical, and physical methods has been proposed for intracellular delivery.^[^ ^88^ ^]^ For instance, when cells “ride” through flow‐mediated confinement devices, they develop transient pores in their plasma membrane, allowing exogenous factors to enter the cytoplasm without endocytosis. Taking advantage of this “mechanoporation”, versatile flow‐mediated confinement device have been developed to deliver a wide variety of materials, including gold nanoparticles, carbon nanotubes, quantum dots, proteins, siRNA oligomers, and polysaccharides to diverse populations of cells, including primary fibroblasts, embryonic stem cells, and a range of immune cells, all at a blazingly fast rate of 20 000 cells s^−1^.^[^ ^86^ ^]^ Building on this, successive studies demonstrated that this microfluidics‐based approach preserved the functionality of primary immune cells and could enable antigen presentation by immune cells to elicit a CD8+ T cell response for cancer cell therapy.^[^ ^89^ ^]^ In a Phase I clinical trial, this approach required less than two weeks to successfully engineer primary immune cells before being administered back into patients, which was well tolerated without any dose‐limiting toxicity.^[^ ^90^ ^]^ Other studies have aimed to improve the efficiency of this technology via the addition of an electric field‐mediated, reversible nuclear membrane disruption step to enhance the nuclear delivery and rapid expression of DNA in cells at high throughput. This disruption‐and‐field‐enhanced delivery approach was able to support co‐delivery of DNA, RNA, and protein, which was not possible via other delivery techniques.^[^ ^91^ ^]^ Remarkably, confinement‐mediated cell deformation and the resultant transient plasma membrane pores have also been shown to enhance the diffusion of macromolecules *out* of cells. Specifically, flow‐mediated confinement has been utilized to enhance their secretion of extracellular vesicles, allowing for optimized collection for downstream applications.^[^ ^92^ ^]^

#### 3.1.2 Flow‐Mediated Confinement Devices for Advanced Cell Reprogramming

Cellular reprogramming of human cells into iPSCs or other valuable cell types has enormous potential for regenerative medicine, personalized therapies, disease modeling, and drug screening.^[^ ^93^ ^]^ However, current protocols relying on chemical or biological factors suffer from poor reprogramming efficiency and low throughput. Recently, it was shown that confinement‐induced nuclear deformation causes chromatin reorganization, making them more accessible, and that this can be optimized to improve the efficiency of cell reprogramming.^[^ ^84b^ ^]^ When cells were flowing through confining microchannels 15 µm high and 7 µm wide, they experienced an active mechanical loading that caused transient nuclear deformation without compromising cell viability. This millisecond‐scale deformation, when introduced during early stages of fibroblast reprogramming, boosted neural transdifferentiation eight‐fold. A detailed investigation into the mechanism revealed that this nuclear deformation caused nuclear envelope reorganization, lamin A/C disruption, detachment of heterochromatin from nuclear lamina, and down‐regulation of both the heterochromatin marker H3K9me3 and overall levels of DNA methylation, proving that mechanical stimuli can overcome the epigenetic barrier of heterochromatin and greatly improve reprogramming efficiency. This confinement‐based mechanopriming was also effective in fibroblast dedifferentiation to iPSCs and the neural transdifferentiation of macrophages, proving that the approach can be utilized for diverse cell engineering applications (Figure 2). Specifically pertinent to cell manufacturing, the microfluidic device was shown to be straightforward to scale up, allowing for conditioning and collection of cells at a high rate and underscoring its potential for real‐world applications.^[^ ^84b^ ^]^

#### 3.1.3 Confinement‐Mediated Mechano‐Phenotyping of Cells

In addition to their direct application in cell engineering, flow‐mediated confinement devices offer an inexpensive and label‐free platform to stratify cells based on cellular deformability, which has been shown to vary as a function of cell type.^[^ ^94^ ^]^ The combination of microfluidics and deep learning to quantify cellular deformability allowed for the analysis of 25 000 cells min^−1^ without sacrificing sensitivity. This device‐and‐algorithm approach was validated across multiple cancer types, yielding an inexpensive platform to mechano‐phenotype cancer cells and providing new possibilities for cell sorting, liquid biopsy analysis, and drug screening (Figure 2).^[^ ^95^ ^]^

### 3.2 Self‐Driven Confinement Devices

Self‐driven confinement devices are not as high throughput as flow‐mediated confinement devices due to the much longer period of time it takes cells to “crawl” through the confining microchannels. During this process, cells have been found to undergo rapid nuclear and cell body deformations,^[^ ^96^ ^]^ cytoskeletal remodeling,^[^ ^97^ ^]^ shifts in migratory mode,^[^ ^85a^ ^]^ chromatin reorganization,^[^ ^98^ ^]^ and in some cases, nuclear envelope rupture.^[^ ^99^ ^]^ Self‐driven confinement devices have thus far been primarily used as investigatory platforms to study cell migration dynamics in various physiological and pathological conditions. However, owing to their relative gentleness compared to flow‐mediated confinement devices, as well as the significant impact they have been found to have on various critical cellular processes, they have great potential for cell engineering (Figure 2).

#### 3.2.1 Self‐Driven Confinement Devices for Regulation of Stem Cell Fate

Given their importance for personalized cell therapy and regenerative medicine, the ability to study and modulate stem cell fate in a controlled fashion is a crucial goal for cell manufacturing.^[^ ^59c^ ^]^ In the body, stem cells have been found to migrate through dense confining matrices, supporting the notion that self‐driven confinement devices are more physiological than rapid flow‐mediated confinement devices.^[^ ^100^ ^]^ However, the field's understanding of self‐directed confined migration's role in directing stem cell fate is still preliminary. One early investigation found that when human bone marrow‐derived stem cells migrated through 3 µm wide transwell assay pores, they exhibited signs of nuclear damage but also showed enhanced osteogenic differentiation signaling.^[^ ^101^ ^]^ Similarly, a recent study shows that stem cells undergo significant nuclear deformation during their active migration through 3 µm wide channels in a self‐driven confinement device, which induced chromatin reorganization via significant H3K9 acetylation. This change in genome regulation promoted osteogenic differentiation of stem cells post‐confinement.^[^ ^102^ ^]^ Thus, self‐driven confinement devices possess immense potential to engineer stem cell fate. Similarly, a recent study using self‐driven confinement devices showed that the migratory potential of T cells and CAR T cells is influenced by mechanical confinement.^[^ ^103^ ^]^ This suggests that mechanical pre‐conditioning of these cells using self‐driven confinement devices could be harnessed to improve their trafficking into solid tumors. However, the low‐throughput nature of these devices currently limits their application in large‐scale stem‐cell engineering. Future studies aiming to develop high throughput self‐driven confinement devices will likely aid in the development of confinement‐mediated strategies for stem‐cell engineering.

#### 3.2.2 Other Applications of Self‐Driven Confinement Devices

In addition to their limited but growing utilization in cell engineering, self‐driven confinement devices have been deployed for a number of biotechnology applications. As these devices are popular in vitro models of confined cell migration under normal and pathological conditions, they can be used as high‐throughput tools for diagnostics, prognostics, and disease modeling.^[^ ^104^ ^]^ A self‐driven confinement device taking advantage of the spontaneous migration of primary neutrophils isolated from patients with major burns was able to aid in the diagnosis of sepsis two days earlier than standard tests. By quantifying the neutrophil migration phenotype with high precision, it was also able to distinguish between patients developing sepsis and those who were not.^[^ ^105^ ^]^ Similarly, a microfluidic assay for quantification of cell invasion (MAqCI) based on self‐driven confinement devices could profile patient‐derived cancer cell migration and proliferation. The patient‐specific profile could successfully quantify the metastatic propensity of cancer and also predict patient‐specific clinical outcomes with high efficiency at low cost.^[^ ^106^ ^]^ Another recent work utilized a self‐driven confinement device to screen 166 potential antimetastatic cancer drugs from a novel mechanoreceptors compound library, identifying three compounds that significantly inhibited confined migration of cancer cells^[^ ^107^ ^]^ and highlighting how devices assaying confined migration provide a more physiologically relevant environment than standard 2D migration assays.

### 3.3 The Future of Confinement Devices for Mechanobiological Control of Cells

#### 3.3.1 Toward Physiologically Relevant Multicue Devices

In just over a decade, PDMS‐based microfluidic confinement devices have evolved and matured to become powerful tools for cell engineering. Their impact can be further augmented by their flexibility and compatibility with a wide variety of possible biophysical/chemical cues (discussed in Section 2). For example, cells in vivo experience hydrostatic pressure and fluid shear stress, which have been shown to be important drivers of mechanosensitive cell behavior.^[^ ^108^ ^]^ Microfluidic platforms are promising tools to simulate these mechanical microenvironments, given their precise control over pressure and flow rate.^[^ ^109^ ^]^ Microfluidic systems, coupled with advanced micro‐ and nanoscale surface modifications, may emerge to present cells with patterned biochemical cues and nanotopographies in conjunction with confinement to drive cells toward a desired state.^[^ ^110^ ^]^ Such physiologically relevant multicue devices may offer superior control over cell state and significantly enhance the efficiency of cell engineering.

#### 3.3.2 Toward Scalable Cell Manufacturing Platforms

With existing advances in microfluidics and our expanding knowledge of confinement mechanobiology, flow‐mediated and self‐driven confinement devices have the potential to play a large role in cell manufacturing. Microfluidic devices are highly compatible with real‐time optical imaging and downstream multi‐omics analysis, enabling high‐throughput tracking and analysis of confinement‐induced cellular and subcellular adaptations.^[^ ^98^, ^111^ ^]^ Beyond this, microfluidic confinement devices can easily incorporate elements of automation and scalability via parallel microchannel fields and 3D feature design, greatly enhancing cell manufacturing efficiency and reducing cost.^[^ ^84b^ ^]^

Taken together, the physical confinement of cells, induced using versatile PDMS‐based microfluidic tools, represents a new and exciting paradigm that cell engineers can and should utilize to more effectively control cell manufacturing outcomes.

## 4 Multiscale Engineering Interfaces for Immune and Stem Cells

### 4.1 Challenges and Opportunities in Cell Engineering

Engineering immune and stem cells has become a cornerstone of next‐generation cell‐based technologies. Engineering cellular immunotherapies often involves the isolation of autologous immune effector cells, such as T cells or NK cells, followed by ex vivo genetic modification using viral or non‐viral vectors, enhancing their cancer‐targeting capabilities. In the case of T cells, this is most commonly achieved by introducing either a CAR or a transgenic T cell receptor (TCR), both of which reprogram the cells to recognize and eliminate cancer‐associated antigens.^[^ ^112^ ^]^ CAR‐based technologies have been successfully applied to numerous immune cell types such as T cells, NK cells, and macrophages, each presenting unique therapeutic opportunities and engineering challenges.

Current manufacturing workflows for CAR‐based immunotherapies, particularly CAR T, and the emerging CAR NK and CAR macrophage platforms, generally involve four key stages: (i) isolation of primary immune cells or their precursors, (ii) cell activation or differentiation, (iii) genetic modification with CAR constructs, most often via viral vectors or electroporation (EP), and (iv) expansion or preparation for reinfusion. Among these, transfection remains the most critical and technically challenging step, directly influencing the functionality, safety, and scalability of the final product.

In clinical‐scale production, EP remains the most widely used *non‐viral* method for gene delivery into immune cells, yet it is increasingly recognized as suboptimal, particularly when delivering large or complex CAR constructs. It suffers from inefficient nuclear delivery, high cell toxicity, and poor functional yields of viable, potent CAR‐expressing cells.^[^ ^113^ ^]^ These limitations are further exacerbated in more fragile or less permissive cell types, such as NK cells and macrophages, which show lower transfection efficiencies and greater sensitivity to electroporation‐induced stress.^[^ ^114^ ^]^ Viral vectors, while still dominant in clinical manufacturing, present their own challenges: they are constrained by limited cargo capacity, are costly to produce at scale, and raise regulatory and safety concerns, especially when applied to emerging CAR‐NK and CAR macrophage therapies that require flexible, transient, or multiplexed genetic programs.^[^ ^115^ ^]^

In contrast to immune‐cell engineering, stem cell‐based approaches present a different set of challenges and engineering requirements. While immune cells are genetically modified to enhance cytotoxicity^[^ ^116^ ^]^ and specificity or to mitigate excessive T‐cell activation,^[^ ^117^ ^]^ stem cells must be engineered to control self‐renewal, lineage commitment, and functional integration, often requiring precise, non‐integrative delivery of genetic or epigenetic modulators (e.g., mRNA, episomal vectors) to maintain cellular identity and safety.^[^ ^118^ ^]^ Stem cells hold exceptional therapeutic promise due to their capacity for self‐renewal and differentiation into specialized cell types, supporting applications in personalized disease modeling, tissue repair, and organ regeneration.^[^ ^119^ ^]^ However, engineering stem cells in a safe, efficient, and clinically relevant manner remains a major challenge. Conventional delivery methods, such as viral vectors and EP, can compromise genomic integrity,^[^ ^120^ ^]^ reduce viability,^[^ ^118^ ^]^ and induce epigenetic instability,^[^ ^121^ ^]^ limiting their translational potential.

Addressing these limitations requires the development of next‐generation bioengineering platforms. Leveraging on the mechanobiology principles mentioned in biomaterial‐based (Section 2) and confinement‐driven (Section 3) strategies, we highlight recent advances in microscale and nanoscale engineering strategies that offer direct, interface‐level control over immune and stem cell behavior, enabling precise manipulation of membrane curvature, force transmission, and delivery efficiency.

### 4.2 Nano‐ and Microscale Routes for Immune‐Cell Engineering

**Figure 3:**
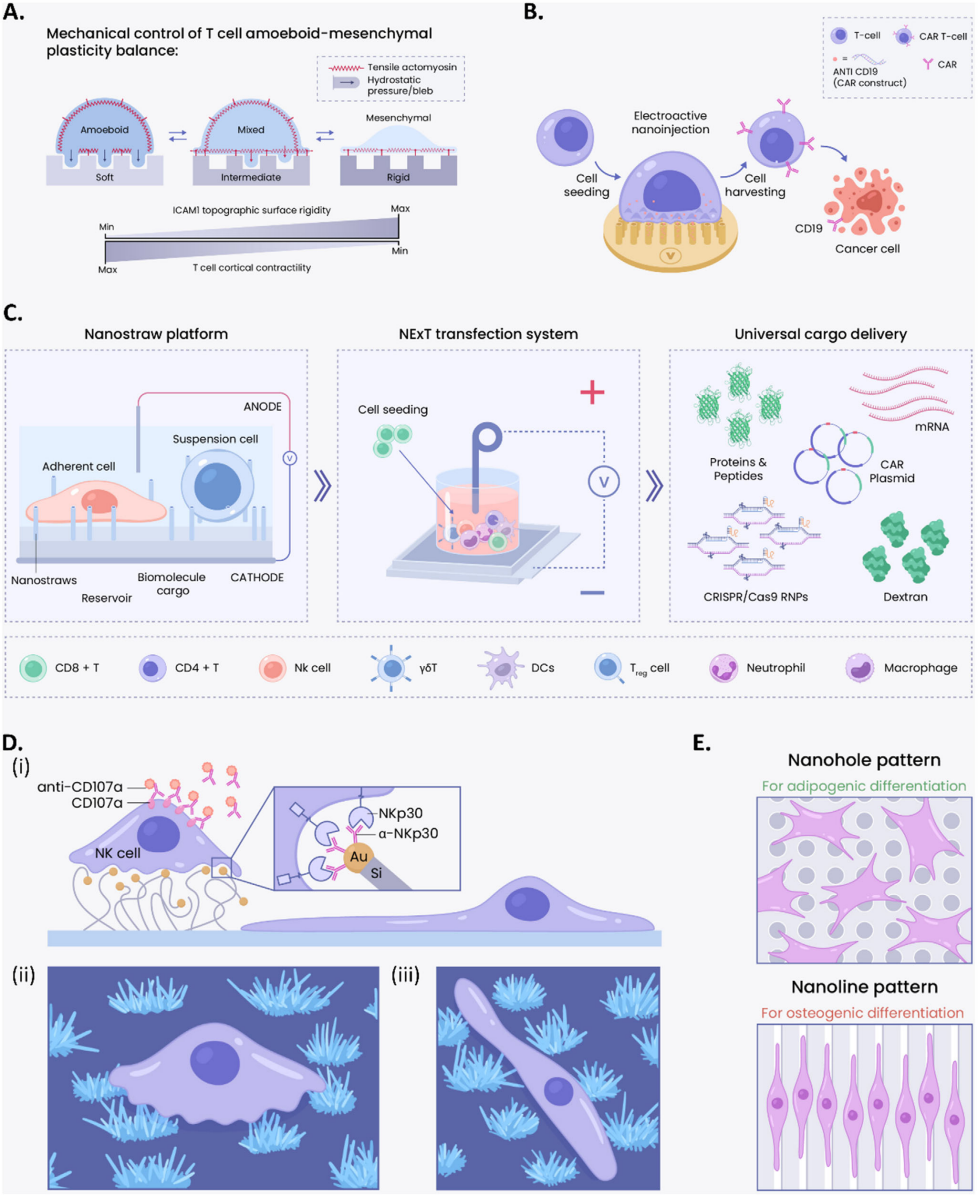
Nanoscale T cell engineering devices: A) Mechanically controlling the mesenchymal‐to‐amoeboid transition of T cells. Substrate stiffness has a profound impact on T cell morphology and migration dynamics. Softer substrates direct T cells toward an amoeboid state, whereas stiffer substrates lead to mesenchymal morphology in T cells.^[^ ^122^ ^]^ Adapted under the terms of the Creative Commons Attribution 4.0 International License (CC BY 4.0). Copyright 2021, The Author(s). B) Electroactive nanoinjection for T cell engineering. Nanotubes allow CAR constructs to be delivered into T cells at low voltages, enabling the generation of CAR T cells and their subsequent killing of cancer cells.^[^ ^123^, ^124^ ^]^ C) NExT technology for non‐viral high‐throughput genetic engineering of primary immune cells. The approach uses high‐aspect‐ratio nanostraws and electric fields for precise intracellular delivery, achieving efficient intracellular delivery of diverse biomolecules while minimizing cell perturbation and cytotoxicity.^[^ ^125^ ^]^ Reproduced with permission. Copyright 2025, Elsevier. D) NK cell‐activation assisted by nanowire forests. (i) illustrates the chemical (nanowire‐tip functionalization) and physical (nanowire forests and smooth surfaces) stimuli on the substrate and the different morphologies of NK cells on the two surfaces. (ii) and (iii) on the right show the interaction between NK cells and nanowire forests and smooth surfaces, respectively.^[^ ^126^ ^]^ E) The FANTAs platform for inducing multi‐lineage differentiation.^[^ ^127^ ^]^

Numerous strategies have been developed to engineer immune cells, particularly T cells, to enhance the efficacy of cell‐based therapies against hematological malignancies and solid tumors.^[^ ^6^ ^]^ In this sense, many designs for nano‐ and microscale platforms have been explored. Among **nanoscale routes**, engineered nanotextured elastic substrates have been demonstrated to be a breakthrough platform. By mimicking the structural and mechanical features of 3D tumor environments, optimized parameters for enhancing CAR T cell motility can be determined. To analyze the architectural and mechanical regulation of T cell phenotypic plasticity and cell migration, “2.5D” nanotextured (grooves/ridges widths  =  800 nm) PA gel surfaces were fabricated with varying stiffnesses. These substrates displayed Hookean elasticity with shear moduli of G'  =  2.3, 8.6, 16, 50, and >1000 kPa. Surfaces were then functionalized with either ICAM1 (intercellular adhesion molecule 1) or ECM proteins. This setup allowed the authors to examine how different mechanical environments influence T‐cell behavior and migration patterns. On stiffer nanotextures (G’ = 50 kPa or greater, **Figure** **3**A), T cells displayed more random migration with reduced directionality along nanogrooves. This environment enhanced mesenchymal‐like spreading, characterized by an elongated shape due to lower cortical contractility, leading to reduced migration efficiency. Strategic disruption of T‐cell microtubules (using nocodazole) has been shown to increase Rho‐mediated contractility (critical in regulating cellular mechanical properties), which in turn enhances T‐cell motility and capacity to infiltrate through the complex and stiff structures typical of 3D tumor microenvironments, ultimately exerting cytotoxic effects.^[^ ^122^ ^]^ The implications are significant for the application of CAR T cells in cancer therapy, further highlighting how mechanobiology can be leveraged to enhance CAR T‐cell efficacy.

A key challenge in T‐cell engineering is to deliver cargo to cells with high efficiency and low cytotoxicity. To achieve this, a wide variety of physical approaches have been designed to penetrate, disrupt, and perturb the T‐cell plasma membrane.^[^ ^118^, ^128^ ^]^ These include micro/nano injection,^[^ ^123^ ^]^ EP,^[^ ^129^ ^]^ sonoporation,^[^ ^130^ ^]^ laser beams,^[^ ^131^ ^]^ optoporation or photoporation,^[^ ^45^, ^71^ ^]^ and microfluidics—either on their own or in combination with other methods.^[^ ^132^ ^]^ Nanoinjection, which involves the use of nanoneedles, nanowires, nanotubes, or nanostraws for intracellular delivery, offers one of the most direct mechanical interfaces with both individual cells and tissues.^[^ ^133^ ^]^ This close interaction makes both cells and tissues viable targets for therapeutic nanoinjection.^[^ ^2^, ^134^ ^]^ Nanoinjection has been shown to successfully transport materials across the plasma membranes of various cell types, including primary immune cells, in a minimally invasive manner.^[^ ^128^, ^135^ ^]^ It enables efficient intracellular delivery of sensitive cargo, such as gene‐editing tools, nucleic acids, proteins, and other bioactive agents, while preserving their structural and functional integrity.^[^ ^136^ ^]^ As a result, these agents can perform their intended roles inside the cell with high efficiency, supporting high cell viability (often exceeding 90%) and reducing biosafety concerns by eliminating the need for viral vectors or high‐voltage electroporation.^[^ ^136^, ^137^ ^]^ This technique has sparked new advancements in ex vivo cell engineering,^[^ ^123^, ^138^ ^]^ particularly in the production of CAR T cells.^[^ ^123^, ^139^ ^]^

Another efficient approach is to integrate nanoinjection with electroporation to combine the biocompatibility of nanoneedles with the enhanced delivery efficiency of electroporation,^[^ ^140^ ^]^ achieving the best of both worlds. Two emerging techniques, electroactive nanoinjection (ENI) and nanostraw electro‐actuated transfection (NExT) (Figure 3B,C), build upon this approach to enable safe, efficient, and non‐destructive entry into primary T cells.^[^ ^125^ ^]^

ENI combines nanotubes (NTs) with an applied electric field to create a highly localized and uniform electric field at the interface between the nanoneedle and cell membrane, thereby nano‐electroporating the membrane at precise contact points.^[^ ^124^ ^]^ The spatial and temporal control of this electroporation process depends on two key sets of parameters: (1) the topological features of the NTs, which determine the strength, shape, localization, and density of the electric field, and (2) the electric field parameters such as voltage, amplitude, pulse duration, frequency, and waveform which regulate the extent and characteristics of the electroporation. Together, these parameters influence the duration, density, and size of the resulting pores, ultimately enhancing intracellular access, CAR gene delivery, and cell viability. Using vertically aligned electroactive NTs, ENI has demonstrated the ability to nano‐electroporate primary mouse and human T cells with high precision and efficiency. It can deliver a variety of membrane‐impermeable cargo, including antibodies (62.5%), mRNA (55.5%), and plasmid DNA (51.8%), while preserving over 90% cell viability.^[^ ^124^ ^]^ ENI can also be used for gene silencing applications; for instance, it achieved 41.3% silencing efficiency by delivering siRNA targeting the *TRIOBP* gene in GPE86 mouse fibroblasts, underscoring its versatility. In primary human T cells, ENI enabled successful CAR gene delivery (68.7%) and expression (43.3%) with minimal cytotoxicity.^[^ ^123d^ ^]^ Compared to conventional electroporation, ENI achieved nearly three times higher CAR transfection efficiency. Most notably, ENI‐engineered CAR T cells were able to suppress lymphoma cell growth by over 80% in co‐culture experiments.^[^ ^123c,d^ ^]^

The NExT platform enables targeted cytosolic delivery of diverse biomolecules, including proteins, polysaccharides, mRNA, and CRISPR/Cas9 RNPs, with minimal cellular perturbation. NExT achieved up to 94.3% efficiency for BSA‐FITC protein delivery into CD8⁺ T cells, and 93.2% into CD4⁺ T cells.^[^ ^125^ ^]^ It also facilitated the transfection of large molecular weight polysaccharides (e.g., Dextran‐FITC, 70 kDa) with 91.6% efficiency in CD8⁺ T cells. For mRNA delivery, NExT showed 83% mCherry expression in CD8⁺ T cells and 54% eGFP expression in CD4⁺ T cells within 24 h post‐transfection (Figure 3c). The platform has proven effective in performing CRISPR/Cas9‐mediated gene knockouts, achieving 54.2% efficiency for *CXCR4* and 81.9% efficiency for *TRAC* in CD8+ T cells.

A nanoinjection technique called “deterministic mechanoporation” was developed by Basilard BioTech (basilardbiotech.com), a spin‐off from UC Riverside.^[^ ^141^ ^]^ Unlike traditional methods, this approach ensures that each T cell is precisely and individually pierced by a single silicon nanoneedle exactly once and at a defined location. Addressing a key challenge in advancing CAR T‐cell therapies, this controlled and repeatable method of mechanical puncturing offers a more uniform, reliable, and less disruptive means of delivering genetic material. In addition to being an efficient and safe strategy, nanoinjection can also be used as an activation‐free delivery strategy to engineer naïve T cells. Conventional methods such as electroporation, lipofection, or viral transduction typically require prior activation, which compromises the stem‐like phenotype and long‐term persistence of naïve CD8+ T cells. In contrast, a 2024 study described the engineering of naïve T cells with mechanical nanoinjection to address this key bottleneck in immune‐cell engineering.^[^ ^126^ ^]^ The study developed a nanowire array, functionalized with cationic polymers to enable delivery of miRNAs and lentiviruses to resting T cells with >90% efficiency and >90% cell viability, significantly outperforming electroporation (≈30% efficiency, ≈50% viability). Notably, the nanowire interface significantly enhanced the passive uptake of lentiviral vectors by resting T cells, achieving ≈75% transduction efficiency without the need for prior activation, compared to <10% using standard conditions. Furthermore, by co‐delivering a miR‐29 inhibitor (which normally preserves a naïve state) and a miR‐130 mimic (which drives effector differentiation), the authors fine‐tuned T cell fate to promote functional activation while reducing exhaustion markers such as PD‐1. Importantly, nanowire‐engineered T cells conferred improved in vivo protection in mouse models of *Listeria* and influenza, showing the potential of this approach for real‐world therapies. These findings highlight how nanostructured surfaces can safely and efficiently reprogram immune cells without activation, offering a scalable strategy for next‐generation cell therapies.

Beyond T cells, nanoscale platforms have also been applied to modulate the behavior and activation of other immune cell types. In a recent study, nanowires have been shown to mediate functional changes in NK cells.^[^ ^141^ ^]^ The authors interfaced primary human NK cells with silicon chips dotted with forest‐like clusters of randomly oriented thick (99 ± 20 nm) or thin (58 ± 16 nm) silicon nanowires (Figure 3D (i)). The nanowire tips were functionalized with α‐NKp30, an NK‐cell‐activating ligand. Notably, two distinct NK cell morphologies emerged: cells in contact with nanowire forests adopted a *rounded shape* (Figure 3D (ii)), while those on the smooth silicon surface between nanowire forests appeared *elongated* (Figure 3D (iii)). This pattern was replicated on non‐functionalized substrates, confirming that NK cells respond to topographical cues independent of surface chemistry. Morphological differences were associated with functional outcomes: rounded NK cells showed significantly higher activation levels than elongated cells, with thin nanowires producing the most pronounced effect, at a 3.5‐fold increase in the proportion of activated cells. These findings underscore the potential of nanotopographical design to enhance NK cell activation ex vivo and inform the rational engineering of immune‐modulatory surfaces.

In parallel with nanoscale innovations, microscale delivery platforms such as mechanical confinement (as mentioned in section 3), are undergoing rapid refinement and are emerging as highly effective strategies for engineering immune cells. A groundbreaking strategy has led to the development of an innovative microfluidic chip (microbioreactor) designed to produce clinical doses of viable autologous CAR T cells within a compact, automated, and closed‐system environment.^[^ ^143^ ^]^ This method enables the efficient activation, transduction, and expansion of CAR T cells that match the functionality and efficacy of cells produced using traditional systems, while significantly reducing the footprint, space requirements, and reliance on large quantities of cell‐seeding numbers and manufacturing reagents. The microbioreactor demonstrates superior scalability and process intensification, achieving high‐density cell cultures with real‐time monitoring and optimized nutrient supply, ensuring robust CAR T‐cell production. The microbioreactor further shortens production timelines, attaining comparable total T‐cell numbers in just 7 to 8 days, as opposed to the 12‐day culture required by gas‐permeable culture plates. This efficiency, combined with minimal starting cell numbers, significantly lowers the need for isolation beads, activation reagents, and lentiviral vectors per production run. The system requires markedly less medium, up to tenfold lower than larger automated platforms, owing to its ultra‐small 2 mL culture volume (≈100 times smaller than conventional systems). These features cut reagent costs substantially and demonstrate the microbioreactor's potential for scalable, cost‐effective CAR T‐cell manufacturing.

Microfluidic electroporation is another emerging technique with significant potential in CAR T cell engineering. A recent study introduced a continuous‐flow electroporation platform capable of processing up to 256 million cells min^−1^, achieving >95% transfection efficiency for mRNA and >90% cell viability, outperforming traditional cuvette‐based systems.^[^ ^144^ ^]^ This platform was constructed around a microfluidic channel that was either 2 or 10 mm wide and 80 µm deep. The microscale channel height reduces the distance between electrodes, greatly increasing the electric field strength relative to the input voltage; functional electric field strength was obtained at voltages much lower than conventional electroporation. Four days after activation, primary T cells were transfected using the continuous‐flow electroporation platform at voltages ranging from 5 to 29 V to deliver CRISPR/Cas9 ribonucleoproteins (RNPs) targeting the TCR. At 25 V, only 12 ± 2.1% of the treated cells retained TCR expression compared to 89 ± 1.0% in the untreated control group, while maintaining a high viability of 88 ± 1.0%. In contrast, transfection at 29 V resulted in a sharp decline in viability without any meaningful gain in editing efficiency (7.4 ± 1.0% TCR expression), highlighting the importance of voltage optimization to balance gene editing outcomes with cell health. Together, these results highlight the potential of microfluidic electroporation as a scalable, high‐throughput alternative for non‐viral T‐cell engineering, balancing efficiency, viability, and manufacturability.

Most recently in 2025, a study introduced a microfluidic gene delivery platform, termed the Y‐hydroporator, to address long‐standing challenges in allogeneic NK cell immunotherapy.^[^ ^113a^ ^]^ Unlike conventional viral or electroporation‐based approaches, this platform leverages hydrodynamic cell deformation in a Y‐junction microchannel to transiently permeabilize NK cells, enabling efficient intracellular delivery. The device achieved ≈90% transfection efficiency and high viability (>89%) across diverse cargo types, including CAR mRNA and CRISPR/Cas9 RNPs. Notably, the platform enabled generation of anti‐CD19 CAR NK cells with enhanced cytotoxicity and successful knockout of inhibitory NKG2A receptors, improving NK function against HLA‐E‐expressing tumor targets. These results underscore the translational potential of hydroporation for scalable, activation‐free production of advanced NK cell engineering.

Macrophages adapt to their environment through polarization to form two distinct subtypes, M1 (generally pro‐inflammatory) and M2 (generally anti‐inflammatory),^[^ ^145^ ^]^ and controlling this process is key to engineering their immunoregulatory functions for therapeutic purposes.^[^ ^146^ ^]^ Recently, a library of over a million topographies, with dimensions from 100 nm to tens of microns, and varying configurations consisting of nanolines, nanogrids, and hierarchical structures (a combination of micro‐ and nanolines), has been constructed to screen for topographical features that mediate polarization in macrophages.^[^ ^147^ ^]^ Feature patterns that promote either the M1 or M2 phenotype have been successfully identified. Machine learning algorithms based on Gaussian‐process regression were also used to predict the topography‐induced macrophage immunomodulation, yielding a continuous map of macrophage polarization against topographical dimensions. This study exemplified the use of high‐throughput screening and machine learning algorithms to systematically analyze the link between macrophage polarization and the dimensions and configurations of topographies, serving as a blueprint for engineering topographical substrates to develop macrophage‐based immunotherapies.

### 4.3 Nano‐ and Microscale Routes for Stem‐Cell Engineering

As introduced in Section 2, microscale topographic cues influence mechanotransduction pathways and regulate lineage commitment. Here, we discuss recent advances exemplifying these mechanisms with spatially controlled scaffolds. A nanotopographical platform was engineered to enable automated and spatially controlled MSC differentiation. The platform is termed functionally aligned nanoparticle‐trapped nanopattern arrays (FANTAs), and comprises of nanohole and nanoline patterns fabricated via laser interference lithography.^[^ ^131^ ^]^ The authors used UiO‐67‐type metal–organic framework nanoparticles for the extended release of osteogenic differentiation factors by trapping them with the platform. MSCs underwent adipogenesis on nanoholes, and osteogenesis on nanolines with over 98% differentiation selectivity (Figure 3E), resulting in precise spatial control of cell differentiation on a single substrate. The FANTAs offer a high‐efficiency yet inexpensive and easy‐to‐operate nanoplatform for MSC differentiation by combining biophysical cues with prolonged release of biochemical factors. This spatial control enables the modeling of physiological interfaces between distinct cell types, such as osteocytes and adipocytes. The versatility of this platform positions it as a valuable tool for both stem cell–based therapy and disease modeling.

A 2025 study demonstrated that microtopographic cues can direct MSC fate by regulating nuclear tension and chromatin accessibility. Using aligned (1.41 ± 0.47 µm) and randomly oriented (1.30 ± 0.98 µm) microfiber scaffolds, the authors demonstrated that substrate orientation dictates cytoskeletal architecture and nuclear deformation (leading to elongated nuclei on aligned scaffolds and rounded nuclei on random ones), which in turn selectively remodels chromatin accessibility at lineage‐specific loci without altering global chromatin architecture.^[^ ^148^ ^]^ In particular, the aligned topographies induced anisotropic stress and selectively opened chromatin regions associated with neurogenic (*Pax7*, *Nes*), myogenic (*Myod1*), and tenogenic (*Scx*, *Hoxa11*) gene programs by activating the transcription factor TLX. In contrast, random patterns generated isotropic stress that preferentially opened chromatin loci linked to osteogenic and chondrogenic differentiation (*Runx2*, *Osx*, *Bmp2*, *Sox9*), mediated by the transcription factor RUNX2. These chromatin changes were confirmed using ATAC‐seq (assay for transposase‐accessible chromatin using sequencing), which revealed site‐specific alterations, while Hi‐C (high‐throughput chromosome conformation capture) analysis indicated stable higher‐order chromatin architecture. Motif enrichment analysis (a computational method to identify overrepresented transcription factor binding sites in accessible chromatin regions) identified TLX and RUNX as key transcriptional regulators driving lineage‐specific chromatin accessibility in response to mechanical cues. Collectively, the study provides a mechanistic framework linking microtopography‐induced nuclear mechanotransduction to epigenetic reprogramming of stem cells, underscoring the potential of topographic design in regenerative medicine.

### 4.4 The Future of Micro‐ and Nanoscale Innovations for Next‐Generation Cell Therapies

#### 4.4.1 Limitations of Current Delivery Platforms in CAR T Manufacturing

Expanding on the delivery challenges outlined in section 3.4, next‐generation CAR T cell manufacturing requires novel non‐viral, high‐throughput manufacturing platforms capable of multiplexed genetic modifications. The cost of viral vectors and lengthy manufacturing lead times make combining individual CAR T cell products all but impossible, and render Phase 1 trials unattainable for most research centers and start‐up biotech companies. Existing alternatives such as EP are often simple, but high voltages used in EP cause high cell toxicity, low yield, and low functionality.

#### 4.4.2 Innovations Driving CAR T‐Cell Therapy Forward

To clinically improve next‐generation specialized CAR‐based cells, a newer delivery approach is essential to generate CAR T cells using multiple CAR constructs (multiple CARs, selection markers, suicide genes, and fluorescent markers). This will require advanced bioengineering technologies using emerging gene‐editing tools such as precise addition with sequence‐specific targeting elements (PASTE) to more safely facilitate the insertion of larger DNA sequences of up to 36 kb into predetermined genomic sites.^[^ ^149^ ^]^ Such combinations could lead to larger genome modifications or a series of smaller genomic edits (for a candidate gene or set of genes). Ultimately, locus‐specific integration will augment CAR T cell homogeneity and make it possible to engineer versatile products. The emerging micro‐ and nanoscale routes are in the process of positioning themselves to address some of those challenges. Such a fundamental advance in designing safe and effective non‐viral technologies will have major potential as a new platform for future CAR T‐cell therapies.

## 5 Clinical Challenges of Advanced Cell Engineering and Manufacturing

### 5.1 Rising Demand for Scalable Cell Engineering

Cell‐based technologies such as CAR‐based immunotherapies and stem cell‐based regenerative medicine are rapidly expanding, driven by growing clinical demand and fundamental and technological innovation. Over 1000 active clinical trials are currently exploring CAR T therapies, with 40–50 products projected to reach market approval by 2030.^[^ ^150^ ^]^ Parallel to CAR‐based immunotherapies development, stem cell therapies, especially those based on iPSCs and MSCs, are also gaining clinical traction. As of 2025, 115 iPSC‐based clinical trials have been approved,^[^ ^151^ ^]^ targeting a wide range of degenerative diseases, with many more trials involving adult stem cells underway.^[^ ^152^ ^]^ Much like immune cell therapies, the advancement of stem cell–based approaches requires continued innovation in intracellular delivery, reprogramming strategies, and emerging micro‐ and nanoscale technologies for precise cell manipulation, culture, and differentiation.

### 5.2 Technical hurdles to cell manufacturing

#### 5.2.1 Scalability and Reproducibility

Despite the promise of advanced engineering strategies for cell manufacturing, including biomaterials, microfluidic confinement platforms, and micro‐ and nanoscale technologies, clinical translation remains a significant bottleneck. Challenges related to scalability and adherence to Good Manufacturing Practice (GMP) standards^[^ ^153^ ^]^ are particularly pronounced. Whether designing biomaterials, fabricating microfluidic devices, or implementing nanoscale tools, batch‐to‐batch consistency in both material and device properties and cellular responses is critical. The more intricate the engineering design, the greater the need for rigorous design controls, risk assessments, and robust quality management systems to ensure regulatory compliance and safety.

#### 5.2.2 Regulatory Hurdles

Regulatory complexity presents additional challenges, particularly when these technologies are incorporated into cell manufacturing processes or used in combination with cells as therapeutic products. Regulatory classification varies across regions. For instance, minimally manipulated autologous stem cells are exempt from full regulatory review in both the United States (FDA) and Australia (TGA).^[^ ^154^ ^]^ However, once combined with materials or devices that modulate cell fate or function, as in the case of engineered matrices, microfluidic stimulation, or nanoscale delivery routes, they often fall under more stringent regulatory frameworks. In Australia, the TGA^[^ ^155^ ^]^ classifies such products as Class 4 biologicals if the cell product is genetically modified (e.g., CAR T cells) or differentiated (e.g., from iPSCs), while MSCs expanded on a non‐differentiating substrate may fall under Class 2 or 3.^[^ ^155^ ^]^ Similarly, the FDA's guidance on “Evaluation of Devices Used with Regenerative Medicine Advanced Therapies; Guidance for Industry” categorizes technologies used alongside regenerative therapies as Class III combination products,^[^ ^156^ ^]^ subjected to the highest level of regulatory scrutiny. These regulations underscore the complexity of demonstrating safety, efficacy, and reproducibility when using advanced bioengineering platforms in clinical applications.

#### 5.2.3 Feasibility

In addition to regulatory and technical barriers, high cost and infrastructure demands further limit the widespread clinical adoption of these technologies.^[^ ^80^, ^157^ ^]^ Manufacturing engineered materials, microfluidic platforms, or micro and nanoscale devices at scale often requires specialized, GMP‐compliant facilities, advanced bioreactor systems, and customized automation workflows – all of which incur substantial capital and operational expenses.^[^ ^143^, ^158^ ^]^ While automation can boost standardization and throughput, it simultaneously increases the financial and technical burden associated with production. Addressing these scalability and cost issues is essential for translating bench‐scale innovations into widely accessible clinical solutions.

#### 5.2.4 Toward Practical Translation

Despite these challenges, clinical translation can be accelerated through the strategic use of engineering platforms that already possess regulatory approval. Clinically validated platforms such as FDA‐approved collagen scaffolds (e.g., Integra Dermal Regeneration Template), hyaluronic acid hydrogels (e.g., Juvéderm, Synvisc‐One), and PEG‐based systems (e.g., GEM 21S), demonstrate proven safety, scalability, and GMP compatibility, helping to navigate regulatory and manufacturing hurdles. Several cell therapies are already leveraging such materials: MSCs have been delivered in clinical trials using hydrogels to enhance retention and paracrine signaling,^[^ ^159^ ^]^ while immune cell therapies, including CAR T cells, are being explored within collagen or fibrin matrices to improve local persistence and reduce systemic toxicity (e.g., NCT04007029).^[^ ^160^ ^]^ The ViaCyte PEC‐Encap macroencapsulation system, used to deliver stem cell‐derived pancreatic progenitors,^[^ ^161^ ^]^ further demonstrates how regulatory‐cleared devices can facilitate both immune protection and functional integration. Collectively, these examples highlight the translational potential of advanced cell engineering strategies as scalable, safe, and effective platforms for next‐generation cell engineering and manufacturing.

### 5.3 Other Emerging Physical Modalities for Mechanical Cell Engineering

In addition to the mechanical modalities discussed in Sections 2, 3, 4, a number of emerging physical technologies are used to stimulate desired cell behaviors, including ultrasound^[^ ^162^ ^]^ magnetic fields,^[^ ^163^ ^]^ vibration,^[^ ^164^ ^]^ gravitational/buoyancy effects,^[^ ^165^ ^]^ and emerging nanofabrication routes.^[^ ^166^ ^]^ These innovative approaches offer unique opportunities to precisely control cellular processes in ways that complement or extend traditional mechanobiological strategies. Ultrasound, for example, can deliver non‐invasive mechanical stimuli at varying frequencies to enhance cell differentiation,^[^ ^167^ ^]^ proliferation,^[^ ^168^ ^]^ and drug uptake.^[^ ^169^ ^]^ Similarly, magnetic fields can be used to manipulate cells,^[^ ^9a^ ^]^ enabling spatial organization, targeted delivery,^[^ ^170^ ^]^ or the creation of mechanically responsive scaffolds.^[^ ^171^ ^]^ Since cells in the body are attached to ECM via focal adhesions, vibration, considered oscillating mechanical load often used at high frequency and low amplitude, can be transmitted to cells via mechanosensation and mechanotransduction.^[^ ^164^ ^]^ Gravitational and buoyancy effects, particularly in microgravity environments, are increasingly recognized for their ability to modulate cellular morphology and function.^[^ ^165^, ^172^ ^]^ By integrating these advanced tools with existing mechanobiological frameworks, researchers are refining cell engineering strategies to achieve more precise, scalable, and reproducible outcomes, paving the way for next‐generation cell‐based therapies and manufacturing.

### 5.4 Learning from Japan to Accelerate the Path to Clinical Readiness

Japan's experience offers a compelling case study in how national investment, fast‐track regulatory frameworks, and manufacturing innovation can bring cell‐based therapies closer to clinical impact.^[^ ^173^ ^]^ With over 60 active iPSC trials and the emergence of AI‐guided robotic manufacturing systems, Japan is demonstrating how scalable platforms can support both personalized and allogeneic treatments for conditions such as macular degeneration, Parkinson's disease, and spinal cord injury. Notably, the transplantation of iPSC‐derived retinal strips illustrates how patterned, sheet‐like cell constructs—requiring precise dimensional control and spatial placement—may benefit from microfabrication‐based approaches. The ability to engineer implantable cell assemblies at microscale resolution opens new possibilities for minimally invasive delivery and integration into host tissues, extending the role of microfabrication from in vitro platforms to therapeutic deployment. Yet challenges remain around genomic safety, scalability, and consistent clinical efficacy. Future strategies could benefit from an integrated, combinatorial approach that couples mechanistic insight, automation, and regulatory alignment with advances in micro‐ and nanotechnology, ultimately unlocking scalable, precise, and globally impactful cell‐based technologies.

## 6 Conclusion

Taken together, the integration of advanced engineering tools—from biomaterials and microfluidics to physical stimulation and gene delivery—points toward a new era in precision cell manufacturing. However, realizing this potential will require coordinated efforts in design, standardization, and regulatory navigation. Strategic adoption of already‐approved platforms, combined with global case studies such as Japan's, may serve as blueprints for accelerating safe and scalable clinical translation.

## Conflict of Interest

The authors declare no conflict of interest.

## Data Availability

The data that support the findings of this study are available from the corresponding author upon reasonable request.
